# Marine Sponge Derived Natural Products between 2001 and 2010: Trends and Opportunities for Discovery of Bioactives

**DOI:** 10.3390/md12084539

**Published:** 2014-08-19

**Authors:** Mohammad Ferdous Mehbub, Jie Lei, Christopher Franco, Wei Zhang

**Affiliations:** 1 Centre for Marine Bioproducts Development, Flinders University, Adelaide, SA 5042, Australia; E-Mails: mohammad.mehbub@flinders.edu.au (M.F.M.); etuferdous@yahoo.com (J.L.); 2 Department of Medical Biotechnology, School of Medicine, Flinders University, Adelaide, SA 5042, Australia; 3 Department of Fisheries Technology, Faculty of Fisheries, Hajee Mohammad Danesh Science and Technology University, Dinajpur 5200, Bangladesh

**Keywords:** marine sponges, porifera, marine natural products, anticancer, sponge-associated bacteria, drug, bioactive compounds

## Abstract

Marine sponges belonging to the phylum Porifera (Metazoa), evolutionarily the oldest animals are the single best source of marine natural products. The present review presents a comprehensive overview of the source, taxonomy, country of origin or geographical position, chemical class, and biological activity of sponge-derived new natural products discovered between 2001 and 2010. The data has been analyzed with a view to gaining an outlook on the future trends and opportunities in the search for new compounds and their sources from marine sponges.

## 1. Introduction

Discovery of marine derived natural products is a promising, but comparatively new field, which started with the discovery of unusual nucleoside derivatives in the sponge *Tethya crypta* in the 1950s by Bergmann and Feeney [1,2]. In the early 1960s, research on marine natural products was driven by chemical studies and few compounds were tested for any relevant bioactivity [3] such as production of a pyrrole antibiotic by a marine bacterium *Pseudomonas bromoutilis* [4]. However, utilization of marine organisms as sources of bioactive metabolites started seriously at the end of 1960s [5] with the isolation of prostaglandin derivatives from the Caribbean Gorgonian *Plexaura homomalla* [6]. In the 1980s effective collaborations were established between marine chemists and pharmacologists and the investigations were focused on central nervous system membrane active toxins, ion channel effectors, anticancer and anti-viral agents, tumor promoters and anti-inflammatory agents [7]. In the 1990s pharmaceutical and biotechnological industries focused their screens on chemical libraries of both natural products, as well as synthetic compounds produced by combinatorial methods [8]. Invertebrates, mainly sponges, tunicates, bryozoans or molluscs provided the majority of the marine natural products involved in clinical or preclinical trials [9].

The discovery of marine natural products has accelerated over the last two decades with the number of new compounds discovered annually increasing from 20 to more than 200 [10]. It has been estimated that by 2010 more than 15,000 new marine natural products (NMNP) had been discovered [11,12,13] with 8368 new compounds recorded for the decade between 2001 and 2010. This constitutes over half of all the compounds discovered since 1951.

**Figure 1:**
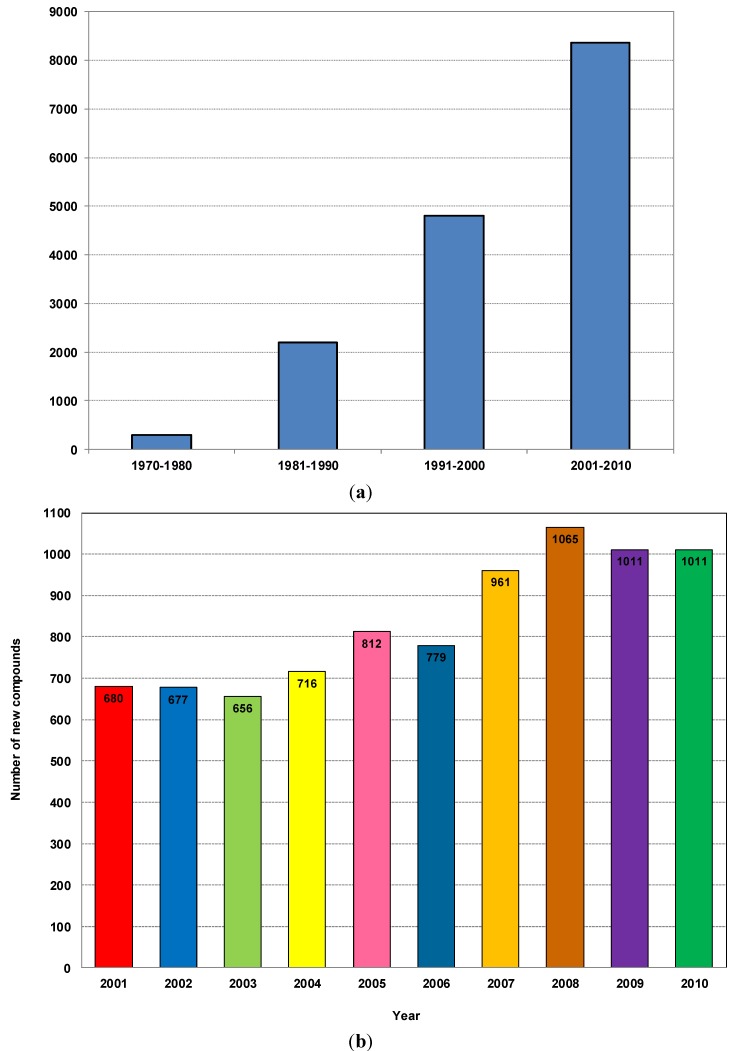
(**a**) Number of new compounds isolated from marine organisms per decade from 1970 to 2010; (**b**) Total number of new compounds isolated from different marine organisms from 2001 to 2010.

Among all the marine organisms investigated, marine sponges (Porifera) are recognized as the richest sources of NMNP, with about 4851 compounds to date, contributing to nearly 30% of all marine natural products discovered so far. It should be noted that of these, 1499 new compounds were isolated in the five years from 2008 to 2012 [14,15,16,17,18]. This makes sponges the most prolific marine producers of compounds with more than 200 new compounds reported each year for the last decade [19]. With this myriad of NMNP available, numerous studies have revealed a broad spectrum of biological activities for these compounds, including anticancer, antiviral, antibacterial, antifungal, antiprotozoal, anthelmintic, anti-inflammatory, immunosuppressive, neurosuppressive, neuroprotective, antifouling and a range of other bioactivities [20]. In addition, as infectious microorganisms evolve and develop resistance to existing pharmaceuticals, marine sponges provide novel leads against bacterial, fungal and viral diseases [19,21]. Figure 1a shows the almost linear growth of new compounds over the last three decades. It is predicted that if this rate can be sustained, the discovery of marine natural products from sponges, in particular, and as well as other major marine organisms will bring about new and effective therapies against human diseases [22,23,24].

Figure 1b shows the trends of novel marine natural products discovered from different phyla of marine organisms during 2001–2010. The annual discovery of marine natural products remained at a constant level of about 500 products in the late 1990s [10] but this number has increased from 600 to over 1000 compounds from 2008 to 2010, a significant increase which was partly driven by new developments in modern analytical technology and instruments, especially the development of the high resolution nuclear magnetic resonance spectrometer (NMR) and mass spectrometry (MS) coupled with high-performance LC and GC [10].

Although sponges have shown the highest potential for natural product discovery, no comprehensive reviews have been published that focus only on compounds from sponges in terms of their source areas, modes of action, chemical class and taxonomy. Two review papers written by Hu *et al*. 2011 and Leal *et al.* 2012 [10,25] described the overall trends in marine natural products, including those from Porifera, during the last two decades. Therefore, we prepared this manuscript based on sponge-derived new natural products from 2001 to 2010 and all the graphs and tables generated for this paper are based on the data reviewed by Blunt *et al.* from the *Natural Product Reports* of 2003–2012 [13,14,15,16,20,26,27,28,29,30]. Information was collected from each individual published paper during this time period and data was generated and analyzed accordingly.

Sponges are exclusively aquatic animals that dominate in many benthic habitats. They are sessile and do not have tissues or sensory organs but have different types of cells which conduct all forms of bodily function. They consume food and excrete waste products within cells without a body cavity [31].

Numerous ecological studies have shown that secondary metabolites produced by sponges often serve defensive purposes to protect them from threats such as predator attacks, microbial infections, biofouling, and overgrowth by other sessile organisms [32,33]. For this reason Porifera are attractive subjects for natural product chemists due to the sheer number of metabolites produced, the novelty of structure encountered, and the therapeutic potential of these compounds in the treatment of human diseases [7]. There is evidence that some compounds originally found in sponge cells are synthesized by microorganisms associated with sponges, since the mesohyl of sponges is often inhabited by microbes and many poriferan natural products resemble metabolites produced by marine microbes [34].

However, these natural products have interesting biomedical potential, pharmaceutical relevance and diverse biotechnological applications [5,35,36,37,38,39]. Moreover, sponge-derived antifouling molecules have been found to be less toxic, environmentally friendly biocides that are often very effective [40].

It is of both scientific and industrial interest as to why and how marine sponges possess such a high diversity of novel marine natural products. As the oldest metazoan, sponges have survived in the ocean for over 600 million years [41] throughout the vast changes experienced by the ocean. The fact that sponges still exist in all waters from fresh to saline, from intertidal to deep-sea, from tropical to frozen waters indicates the tremendous ability of sponges to respond and adapt to the varied environmental conditions over this period. In addition, sponges are one of the most efficient sessile filter feeders: they can filter up to 24 m^3^·kg^−1^ day^−1^ [42]. Bacterial numbers in sponge tissue often exceed those of the surrounding seawater by two to three orders of magnitude as the sponge mesohyl provides a unique ecological niche for particular bacterial species. In many cases, sponge mesohyl harbours the bacterial symbionts (30%–60%) [43]. Bacteria provide their hosts with products of their metabolism, thereby granting the sponge access to bacteria-specific traits such as autotrophy, nitrogen fixation and nitrification [44]. Other examples show that sponge-associated bacteria can process metabolic waste compounds, stabilize the sponge skeleton and provide protection against UV radiation [35,45,46]. The most prominent example of sponge bacterial symbiosis, however, is the involvement of bacteria in the production of bioactive metabolites [47] that have a role in defense [48].

These highly intensive, constant interactions with the environment have given sponges a unique biochemistry to produce the high diversity of metabolites that can either help them survive or prompt them to evolve. Being attached to a solid surface, a sponge is unable to escape when confronted with a predator, and so, when threatened they release stored secondary metabolites that have cytotoxic, antibiotic and feeding deterrent properties [48]. Some chemicals prevent settlement of fouling organisms on the sponge surface and restrict competition for space with neighbors. The sponge bacterial associations and interactions have been widely studied, with evidence that the sponge-associated bacteria can help the sponges to produce secondary metabolites to protect them against their predators [49]. In an ecological context, sponges have developed special mechanisms to protect themselves from pathogenic bacteria, viruses, parasites, fungus and other predators that include both chemical defense mechanisms and physiological responses. Chemical defense mechanisms help to protect sponges against certain deleterious bacteria [33,50,51]. In this way, sponges provide novel leads against viral, fungal and parasitic diseases [52]. By producing different types of toxins or malevolent tastes and odors, sponges protect themselves against predators or inhibit coral overgrowth that could threaten the sponge osculum or other systems. As a physical defense they have spicules and collagen. Sponges may also succumb to microbial and fungal infections which could result in the disintegration of the sponge fibers/tissue and ultimately lead to sponge death [53]. The fact that sponges are susceptible to microbial infection suggests that they should also possess mechanisms to prevent these types of diseases [54]. Maldonado and co-workers [55] showed how sponges recover from a bacterial infection: their ultrastructural study revealed that the sponges secrete successive collagen barriers at the diseased area and abandon decaying body parts external to the barrier.

Recently, the ubiquitous defense enzyme, phospholipase A2 (PLA2) detected in a sponge associated bacterium envisaged the possible functional role in the ecological succession of the host sponge against predatory/fouling pressure in the habitat [56]. In response to predators and pathogens, sponges have engineered complex secondary metabolites from a diverse set of biological precursors. Secondary metabolites are organic compounds that are not directly involved in the normal growth, development or reproduction of organisms. These metabolites produced by sponges and their associated microflora can be classified chemically as alkaloids, terpenoids, glycosides, phenols, phenazines, polyketides, fatty acid products and peptides, amino acid analogues, nucleosides, porphyrins, aliphatic cyclic peroxides and sterols [57,58]. Many of these compounds are very potent because the diluting effect of the ocean drives the construction of molecules that are highly active and stable in saline conditions [59].

Given the significance of sponges in marine natural product discovery, the aim of this review is to present a comprehensive overview of sponge-derived natural product discovery during the recent decade from 2001 to 2010, in order to understand the defining trends and provide insights into avenues for further compound discovery. The temporal trends of the discovery of sponge-derived marine natural products and their biological activities, the sources of discovery in terms of sponge taxonomy, the chemical classes of these natural products, and the countries of collection have been categorized. Our analysis also includes a short description of the relative distribution and contribution of these discoveries with reference to governmental funding, policies and known national priorities given to marine natural products. Finally, the opportunities and challenges have been identified for future R and D in this fast growing field.

The new compounds isolated during the last decade were classified into 18 chemical classes including acid, alkaloid, ester, fatty acid, glycoside, ketone, lipid, macrolide, alcohol, peptide, peroxide, polyketide, quinone, steroid, sterol, terpene, terpenoid and unclassified, based on the data reviewed by Blunt *et al.* from *Natural Product Reports* of 2003 to 2012 [13,14,15,16,20,26,27,28,29,30]. The World Porifera Database [60] was used for the taxonomic classification of the sponges mentioned in the Natural Product Reports [13,14,15,16,20,26,27,28,29,30]. The World Register of Marine Species (WoRMS) database [60] was also used to cross check detailed taxonomical information (order and family) for each surveyed species and to validate and/or update their scientific names [61].

During the recent decade the sponges collected were from 19 known orders as well as 12 sponges of unknown identity which provided new compounds. These included Agelasida, Astrophorida, Axinellida, Chondrosida, Choristida, Clathrinida, Dendroceratida, Dictyoceratida, Hadromerida, Halichondrida, Haplosclerida, Homosclerophorida, Leucosolenida, Lithistida, Lyssacinosida, Ocilosclerida, Poecilosclerida, Spirophorida and Verongida. Sixty two countries (with Antarctica labeled as a country for reporting purposes) were the sources for the sponge samples studied. The bioactivities were mainly classified as anti-Alzheimer’s, antibacterial, antituberculosis, anticancer, antifungal, anti-inflammatory, antimalarial, antiviral and anti-HIV.

## 2. New Compounds and Their Distribution 2001–2010

### 2.1. Yearly Distribution of Phyla that Produce Natural Products Discovered from 2001 to 2010

**Figure 2:**
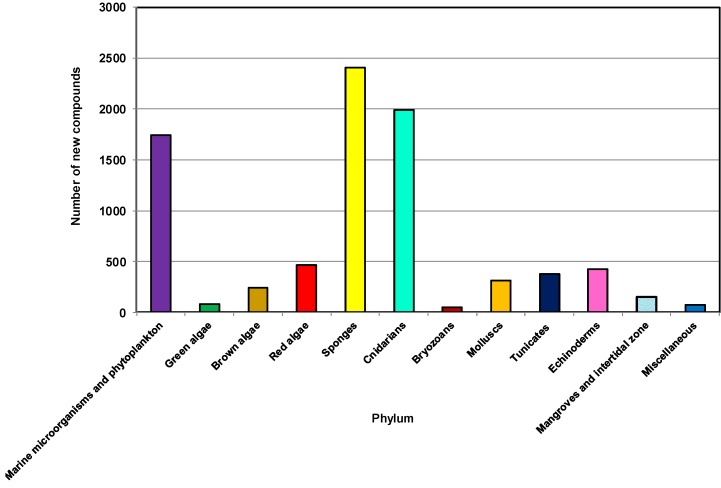
Total number of new compounds isolated from different types of marine sources, 2001–2010.

To investigate the distribution of sources of NMNP, 12 different categories including a separate group comprising marine microorganisms and phytoplankton were used for this review (Figure 2). Invertebrates comprise approximately 60% of all marine animal diversity [62]. Most belong to the phyla Porifera (sponges), Annelida, Arthropoda, Bryozoa, Cnidaria, Echinodermata, Mollusca and Chordata. Several studies addressing marine invertebrates also include these groups of organisms [32,33,63].

As highlighted in other reviews [10,64], the phyla Porifera and Cnidaria have been the two main sources of NMNP. During the last 10 years from 2001 to 2010, more than 2400 new natural products have been discovered from 542 genera and 671 species of sponges. These sponges belong to 19 known orders and 74 families, contributing about 29% of the marine natural products discovered during this decade, making it the largest source among all marine organisms [48,65].

### 2.2. Sponges (Porifera) as a Source of New Natural Products and Drugs for the Future

A review in 2003 collected the most important marine natural compounds which were undergoing preclinical and clinical trials (I, II, III) for anticancer activity. Among those, compounds from sponges were the following: Discodermolide, Hemiasterlins A & B, modified Halichondrin B, KRN-7000, Alipkinidine (alkaloid), Fascaphysins (alkaloid), Isohomohalichondrin B, Halichondrin B, Laulimalide/Fijianolide, 5-Methoxyamphimedine (alkaloid) and Variolin (alkaloid) [66]. A review paper was published by Sipkema *et al.* 2004 about the drugs from marine sponges [67].

Marine natural sources as potential anticancer agents were reviewed in 2011 which mentioned 39 marine-derived potential anticancer agents and among them 18 compounds from sponges with different mechanisms of action [68]. Interestingly, from the 16 marine natural products that are currently under preclinical trials as new drug candidates, most are derived from invertebrates. Of these, Porifera remain the most important phylum, with six of the 16 compounds [69,70,71]. A review paper published in 2013 classified anticancer molecules according to their current status in the clinical phase trials (approved/phase IV/phase III/phase II/phase I) and updated the data to April 2012 [72]. A very recent review, published in 2014, showed the compounds derived from marine sources currently in clinical trials against cancer with more updated information on clinical and late preclinical developments [73]. This paper also mentioned that although many compounds showed potential against cancer and entered clinical trials in cancer, to date, only Cytarabine, Yondelis^®^ (ET743), Eribulin (a synthetic derivative based on the structure of Halichondrin B), and the Dolastatin 10 derivative, monomethylauristatin E (MMAE or vedotin) as a warhead, have been approved for use in humans (Adcetris^®^) [73].

Although a number of compounds from sponges showed promising activity to be potential drug candidates over the last few decades they are generally not ready for further development due to the challenge of obtaining continuous and larger supplies of the compounds, unless they can be chemically synthesized. Considerable quantities of a drug candidate are vital for clinical trials, but only a few milligrams of most natural products can be isolated from marine samples [74]. One solution is farming sponges to source bioactive metabolites [75]. On the other hand, sponge compounds that are produced by sponge-associated microorganisms can be scaled up as the microorganisms are able to flourish independently of the sponge. Another way to ensure supply is using sponge cell culture, although this is still a growing area. Muller *et al.* in 2000 described the production of bioactive compounds by sponge cell culture [76] and Zhang *et al.* in 2003 and Cao *et al.* in 2007 showed improved cell proliferation and spiculogenesis from primmorphs of sponges and dynamics of spicule production in *in vitro* sponge cell culture systems [77]. The sustainable production of bioactive compounds from sponges was reviewed in 2004 [78] and again in 2009 where the advantage and disadvantage of sponge cell culture was discussed, with the conclusion that the understanding of the metabolic pathways is one of the potential advantages of sponge cell culture systems [79]. However, the most promising solution was answered by Wilson *et al.* in 2014; his findings illuminated two promising approaches for addressing the supply problem [80]: firstly, large-scale cultivation of the microorganisms that produce interesting metabolites, and secondly, expressing the biosynthetic pathway of interest in an easily cultivable surrogate host. The discovery of Wilson and Piel and their colleagues identified *Entotheonella* and members of the newly proposed phylum Tectomicrobia as a “biochemically talented” phylum on a par with the actinomycetes [80,81,82]. Thus, these results could facilitate a new era of drug discovery.

### 2.3. The Distribution of New Marine Natural Products from Sponges

To date, about 11,000 species of sponges have been formally described of which approximately 8500 are considered valid, but as many as twice those numbers are thought to exist [83]. Well known sponge fauna in the Caribbean, Mediterranean, and the British Isles each contain 500–800 species, whereas less well characterized sponge fauna in Australia, Papua New Guinea and Indonesia possess a high biodiversity [84]. Sponges are currently divided into four distinct classes, 25 orders, 128 families and 680 genera [59,60].

The sponges reported to produce new compounds in the last ten years were from 19 known orders although a number of sponges were not identified. A careful analysis of the trends of the discovery of new bioactive compounds from different orders of sponges is presented here to guide scientists in future discoveries.

**Figure 3:**
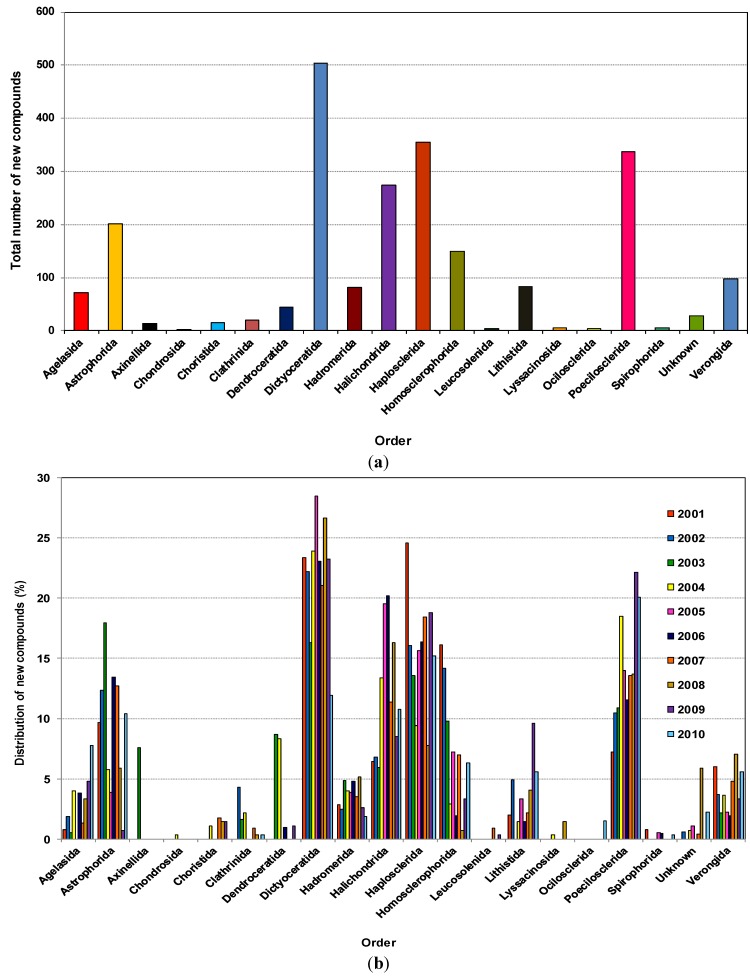
(**a**) Total number of new compounds isolated from different orders of marine sponges 2001–2010; (**b**) Distribution of new compounds isolated from different orders of marine sponges as a percentage found within the year, 2001–2010.

As shown in Figure 3a, 504, 355, 337, 274 and 201 new compounds were found from the five orders Dictyoceratida, Haplosclerida, Poecilosclerida, Halichondrida and Astrophorida, respectively. Notably, these five orders contributed more than 70% of the new compounds. The highest numbers of new compounds were found from Dictyoceratida with 72 found in 2008, 66 in 2004, 63 in 2009, 58 in 2001 and 51 in 2005.

Table 1 shows that some orders have been found to be productive sources of NMNP, many with a large chemical diversity. Astrophorida, Dictyoceratida, Halichondrida, Haplosclerida and Poecilosclerida are the orders from which more than 50 species were studied [83].

Dictyoceratida contributed at least 16% of new compounds each year except in 2010 (11.9%), reaching a peak of 28.5% in 2005 (Figure 3b). Haplosclerida contributed at least 13.58% of new compounds each year except 2004 and 2008 with values of 9.4% and 7.7% respectively. This order yielded the highest number of compounds in 2001 at 24.6%. In the case of Poecilosclerida, a gradual increase was observed until 2004 and reached a peak in 2009 at 22.1%. From 2004 to 2008 Halichondrida contributed at least 11.4% new compounds each year.

This corresponds with a recent review on clinically active compounds from sponges which had Astrophorida, Chondrosida, Dendroceratida, Dictyoceratida, Hadromerida, Halichondrida, Haplosclerida, Lithistida, Poecilosclerida, Spirophorida and Verongida as the main orders from which clinically active compounds were found [85]. Leal *et al.* 2012, covering 1990–2009, found that NMNP were recorded in 17 orders of Demospongiae, and about 89% of the natural products were derived from only eight of those orders, namely Astrophorida, Dictyoceratida, Halichondrida, Haplosclerida, Homosclerophorida, Lithistida, Poecilosclerida and Verongida [25].

**Table 1:** Total number of new compounds isolated from different orders of marine sponges with number of families, genera, species and number of references published from 2001 to 2010.

Order	Number of Families	Number of Genera	Number of Species	Number of References
Agelasida	9	9	21	24
Astrophorida	26	58	63	62
Axinellida	2	2	2	2
Chondrosida	1	1	1	1
Choristida	3	3	3	3
Clathrinida	5	5	7	7
Dendroceratida	7	10	11	13
Dictyoceratida	40	117	145	161
Hadromerida	24	31	32	33
Halichondrida	31	69	86	84
Haplosclerida	52	80	100	120
Homosclerophorida	10	20	39	50
Leucosolenida	1	1	1	1
Lithistida	14	20	23	32
Lyssacinosida	2	2	2	2
Ocilosclerida	1	1	1	2
Poecilosclerida	67	68	81	83
Spirophorida	4	4	4	5
Unknown	8	12	12	10
Verongida	27	29	37	46
Total	334	542	671	741

All NMNP discovered since 1990 were recorded in 64 families belonging to the phylum Porifera. However, about 51% of these products were derived from only nine families: Spongiidae, Dysideidae and Thorectidae in the order Dictyoceratida; Chalinidae and Petrosiidae in the order Haplosclerida; Halichondriidae in the order Halichondrida; Ancorinidae belonging to the order Astrophorida; Plakinidae belonging to the order Homosclerophorida and Theonellidae of the order Lithistida. Here, the highest increase in the number of NMNP annually discovered was recorded for the families Chalinidae and Spongiidae. The family Theonellidae has yielded a number of unique compounds [86] with a broad spectrum of biological activities, including antifungal [87] and cancer cell growth inhibitors [88,89].

**Figure 4:**
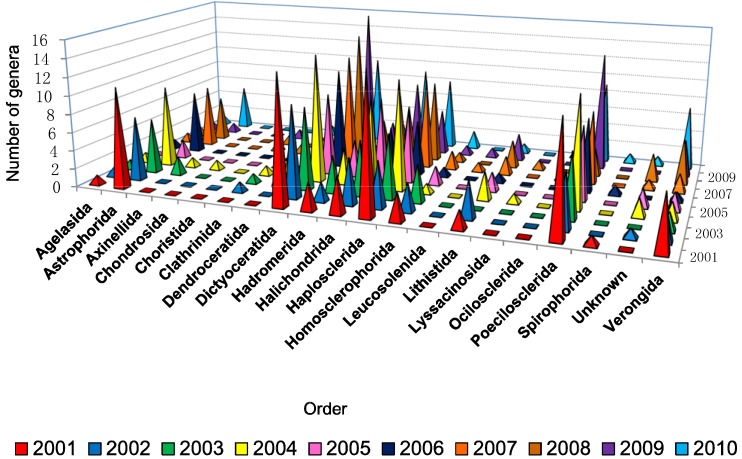
.Number of different types of genera used for isolation of new compounds from different orders of marine sponges from 2001 to 2010.

Figure 4 shows that between 9 and 16 genera were found to produce new molecules each year from the order Dictyoceratida which revealed a high availability of different genera from this particular order, with the highest number found in 2009. This order will be examined in greater detail in a subsequent review covering 2001–2010. Genera belonging to the orders Haplosclerida, Halichondrida, Poecilosclerida, and Astrophorida also showed more than five productive genera for most of the last decade. The number of new compounds correlated with the high diversity of sponges because the higher the diversity the higher the possibility of getting more novel compounds. Another possible reason that makes Dictyoceratida the most prolific producers of NMNP as well as Astrophorida, Haplosclerida, Homosclerophorida and Halichondrida is because these orders harbor high densities of microorganisms [25,85].

### 2.4. Distribution of New Compounds per Species from Different Orders

During the last decade each individual species from different sponges contributed on an average four new compounds under different orders (Figure S1, Supplementary Information). Although very few species have been studied from the orders Axinellida and Choristida, on an average they also produce almost the same number of compounds. This finding permits the inference that each individual species has the potential for contributing similar numbers of new compounds regardless of the order.

### 2.5. Symbiotic Relationships: Sponge Associated Microorganisms

Hentchel *et al.* 2002 reported that although it is generally believed that symbiotic interactions exist between sponges and specific microorganisms, consideration of alternative explanations such as the selective enrichment of ubiquitous seawater bacteria is also important [42]. Moreover, they stated that very specific type of selective pressures, perhaps the hostility to digestion, must exist to establish the uniform composition arrangements of the microbial communities existing in sponges that have otherwise few commonalities [42].

However, it was consequently observed that marine sponges host abundant and diverse communities of symbiotic microorganisms [90]. Webster *et al.* 2012 mentioned that microbial symbionts are undoubtedly important to sponge health, and therefore it is likely that interruptions to symbiosis as a result of climate change/environmental stress will influence sponge health, growth rates or their ability to defend themselves from predation, fouling and disease [91]. Symbiotic interactions between sponges and microorganisms could contribute to sponge nutrition as well [92]. Important roles of the symbionts include photosynthetic carbon fixation [93], nitrification [94,95], nitrogen fixation [44,96,97], and anaerobic metabolism [98]. Another important role of sponge-associated bacteria is the production of potential secondary metabolites, such as antibiotics, antifungal compounds and anti-predation or antifouling compounds [39]. Whilst the microbes associated with the sponges produce the secondary metabolites, possibly all of these sponges have particular microbial associations [99]. More research is necessary to explore the relationship between microorganisms and phytoplankton associated with sponges because it has been suggested that (at least) some of the bioactive secondary metabolites isolated from sponges are produced by functional enzyme clusters which originated from these microorganisms. The role of these microorganisms in sponge biology varies from source of nutrition to mutualistic symbiosis with the sponge [100].

It has been recognized that metabolites synthesized by marine microorganisms associated with sponges could become a major source for the discovery of new drugs, not only because the biological diversity in marine ecosystems like coral reefs or deep sea floors is probably higher than in the rainforest, but because marine microorganisms offer a renewable resource for the scale-up and development of potentially new drugs [101,102].

A number of examples of the functions of the sponge-associated microorganisms are provided to signify the diversity of functions. Dudler and Eberl 2006 conducted a study on interactions between bacteria and eukaryotes which showed increasing evidence to support the hypothesis that secondary metabolites produced by symbiotic bacteria are a result of bacterial cell-to-cell signaling [103]. In a similar vein, Schmidt *et al.* 2008 looked at how organisms cooperate in the synthesis of natural products. They found that partners may exchange and modify the natural products produced by each other and also explained how these secondary metabolites are utilized by the host organisms [104].

Many sponge-derived metabolites resemble bacterial natural products or belong to substance classes typical for these microorganisms [82]. In a recent paper, the Piel group demonstrated beyond doubt that almost all bioactive polyketides and peptides known from the marine sponge *Theonella swinhoei* were attributed to a single phylotype, *Entotheonella* spp. and are extensively distributed in sponges [80].

The diversity in the locations (Okinawa, the Philippines, Indonesia, the Red Sea, Italy, South Africa, and Papua New Guinea) and genera of sponges (*Amphimedon* sp. and *Acanthostrongylophora*) responsible for the production of manzamine alkaloids are widely believed to be a result of a symbiotic relationship between these sponges with common or closely related microorganism(s), which may account for the generation of manzamine enantiomers [105].

*Crambe crambe* (Schmidt, 1862) (Poecilosclerida) is a red incrusting marine sponge present in the Mediterranean Sea and reported to produce diverse PGAs, namely crambescidins 800, 816, 830, 844, as well as isocrambescidin [106,107]. Using 16S rRNA gene pyrosequencing it has been found that the associated bacterial community of *C. crambe* is dominated by a single bacterial species affiliated to the Betaproteobacteria [108].

### 2.6. The Distribution of Chemical Classes

Although acid, ester, ketone, peroxide do not directly fall into chemical classes, we considered these as chemical classes just to show the distribution as such described in the original paper as well as mentioned by Blunt *et al.* [13,14,15,16,20,26,27,28,29,30] in their annual review of marine natural products that were used in preparing this manuscript. In fact, classes of natural products should be classified based on their biosynthesis. However, it is important to remember that a macrolide is often a polyketide and a sterol a steroid and fatty acid belongs to lipid.

**Figure 5:**
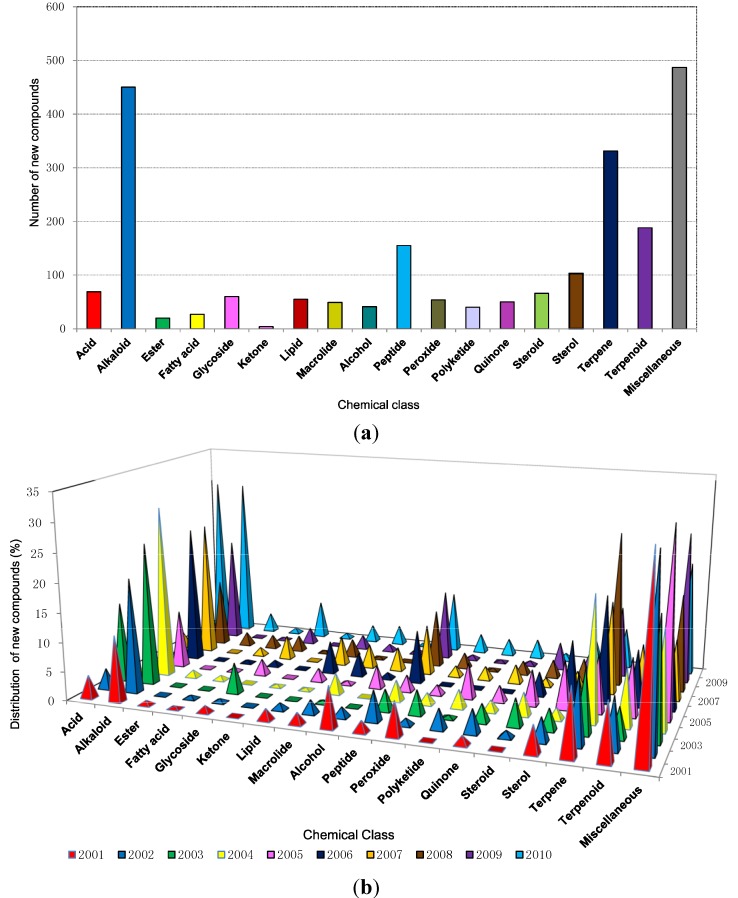
(**a**) Chemical classes of new compounds isolated from marine sponges from 2001 to 2010; (**b**) The distribution of different chemical classes of new compounds isolated from marine sponges from 2001 to 2010.

A wide range of chemical and functional diversity has been observed among new compounds during the last decade. The analyzed data (Figure 5a) has shown that in the last ten years 450, 331, 188 and 155 new compounds were classified as alkaloids, terpenes, terpenoids and peptides, respectively, and these four classes make up approximately 50% of compounds discovered. While alkaloids contributed 20% of the new molecules the terpene/terpenoid classification which are often not readily distinguishable, together made up 23% of the total within the decade.

The chemical diversity of bioactive compounds reportedly produced by sponge-microbe associations showed that certain chemical classes such as quinones, steroids, fatty acids, diketopiperazines, alkaloids, terpenes, terpenoids, trichoverroids and prodigiosin derivatives, diglucosyl-glycerol, polyketides, cyclopeptides, glycoglycerolipid, benzoic acid derivatives are responsible for anticancer or antitumor activity; quinolone derivatives for anti HIV activity; fatty acid esters and fatty acids for anti-inflammatory activity; alkaloids and quinolone derivatives for antimalarial activity; polyketides glycopeptides, α-pyrone derivatives, peptides, proteins, antimycin, lipopeptides, polybrominated biphenyl ether, cyclic depsipetide, terpenes, pentaketides, furan carboxylic acid, alkaloid, diketopiperazine, anthraquinone, chromones, steroid, lactone, quinolone derivative, trisindole derivative, macrolactam, ethers, phenol derivative for antimicrobial activity; and dihydropyridine for neuroprotective activity [85]. In this review it was also evident that anticancer, antimicrobial, anti-HIV, antiflammatory, antimalarial, and neuroprotective disease and antituberculosis were the main classes activity exhibited by most of the new compounds during the recent decade. Terpenoid quinones and hydroquinones, the chemical classes found mainly from Dictyoceratid sponges [11,12,20], have been continuously reviewed and updated. Sesquiterpenoid quinones and hydroquinones showed versatile activities and even a hydro-quinone displays multiple activities [109].

The reason that most sponges produce alkaloids could be for protection from predators such as fish as alkaloids act as good toxicants against predators. Assman *et al.* 2000 presented data suggesting that bromopyrrole alkaloids fulfill multiple ecological functions in the defense mechanisms of the common and diverse genus *Agelas* [110]*.* A striking example of the significance of marine alkaloids for chemical defense against fish is provided by the red coloured sponge *Latrunculia magnificea* (order Poecilosclerida) from the Red Sea. Even though *L. magnificea* growth is exposed, it is apparently avoided by fish, whereas other sponges from the same habitat that are cryptic are readily consumed by fish when artificially exposed [111].

Further information on the different classes of secondary metabolites of marine sponges and their bioactivities are the subject of previous reviews [14,93,112,113].

### 2.7. The Distribution of Bioactive Compounds

**Figure 6:**
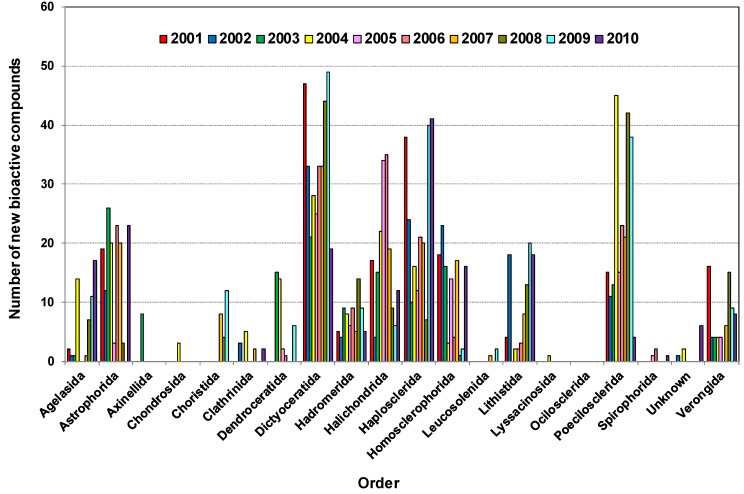
Distribution of bioactive compounds isolated from various marine sponge orders 2001–2010.

Table 2 shows that during the decade until 2010, 332, 229, 227, 173 and 149 new bioactive compounds were found from Dictyoceratida, Haplosclerida, Poecilosclerida, Halichondrida and Astrophorida, respectively, the highest number of new bioactive compounds isolated from different orders of sponges in this period. Dictyoceratida alone contributed 20.6% of all bioactives, Haplosclerida and Poecilosclerida both contributed 14%, whereas Halichondrida and Astrophorida contributed 10.8% and 9.3% respectively. The detail of yearly distribution is presented in Figure 6. Therefore, these five orders contributed 69% of bioactive compounds among all the different orders of sponges.

In sponges the role of the chemical constituents is clouded by the complexity of the sponge-symbiont relationship [114]. The current body of evidence is too limited to make broad generalizations, but it suggests complex chemical and biological interactions that have not yet been resolved [115]. This knowledge will feed into strategies to relieve a major bottleneck for sponge metabolite production, namely: understanding metabolites production in the sponge [79].

**Table 2:** Total number of new compounds isolated from different orders of marine sponges with different bioactivities from 2001 to 2010.

Orders of Sponges	Anti-Alzheimer’s	Antibacterial	Antituberculosis	Anticancer/Cytotoxicity	Antifungal	Anti-inflammatory	Antimalarial	Anti-HIV	Antiviral	Miscellaneous	Total
Agelasida	0	17	0	11	6	0	6	0	0	14	54
Astrophorida	0	8	6	97	7	0	1	5	3	22	149
Axinellida	0	0	0	0	0	0	0	0	0	8	8
Chondrosida	0	0	0	3	3	0	0	0	0	0	6
Choristida	0	0	0	12	0	0	0	0	0	12	24
Clathrinida	0	4	3	3	2	0	0	0	0	0	12
Dendroceratida	0	4	0	14	3	3	0	0	0	14	38
Dictyoceratida	0	38	3	182	11	2	1	5	0	90	332
Hadromerida	0	2	3	45	1	0	0	5	0	18	74
Halichondrida	1	18	4	99	16	1	2	1	0	31	173
Haplosclerida	8	15	2	100	20	0	7	4	0	73	229
Homosclerophorida	0	2	3	55	10	0	9	1	0	34	114
Leucosolenida	0	1	0	2	0	0	0	0	0	0	3
Lithistida	0	5	2	38	9	2	0	16	0	16	88
Lyssacinosida	0	0	0	1	0	0	0	0	0	0	1
Ocilosclerida	0	0	0	0	0	0	0	0	0	0	0
Poecilosclerida	0	17	5	143	21	1	1	4	1	34	227
Spirophorida	0	0	0	4	0	0	0	0	0	0	4
Unknown	0	0	0	2	0	0	0	0	0	7	9
Verongida	0	14	0	17	5	0	0	0	0	34	70
Total	9	145	31	828	114	9	27	41	4	407	1615

The origin and role of a number of compounds such as bioactive peptides within the sponges have yet to be clarified, as many of these compounds have potent activities not always clearly related to their *in situ* role [116]. However, it was subsequently found that antimicrobial peptides (AMPs) are components of innate immunity, forming the first-line of defense used by any organisms against the invading pathogens [117]. Pasupuleti *et al.* 2012 termed AMPs as the key component of the innate immune system [118]. Two good examples of AMPs produced by Porifera are Stylisin and Discodermin A [119,120], although many AMPs have been produced since the first production of Discodermin from sponges [121]. In 2013 a review paper was published on antimicrobial peptides with versatile biological activities which included a few sponges producing AMPs [122].

There is a sign that the quick evolution of molecules related to cell adhesion and pathogen killing (AMP precursors) has been acute in the successful alteration of sponges [123]. In their 2010 review Otero-González *et al.* discussed the new frontier for microbial infection control by antimicrobial peptides including Porifera [121]. A review published by Brogden *et al.* 2005 commented that translocated peptides can modify cytoplasmic membrane septum creation, hinder cell-wall synthesis, constrain nucleic-acid synthesis, inhibit protein synthesis, or obstruct enzymatic activity [124].

Some external activities of these peptides are as antitumorals, antivirals, immunosuppressive and antimicrobial agents, as well as neurotoxins, hepatotoxin, and cardiac stimulants. The various functional roles of some terpenoids are considered as hormones (gibberellins), photosynthetic pigments (phytol, carotenoids), electron carriers (ubiquinone, plastoquinone), and mediators of polysaccharide assembly, as well as communication and defense mechanisms [125]. Terpenes may also act as safeguard for a variety of organisms in the marine world, including algae, sponges, corals, mollusks and fish [33].

It is important to note that most of the orders showed cytotoxicity or anticancer activity although only a few have been tested for *in vivo* antitumor activity. In the five year period from 1986 to 1991, more than 400 novel marine natural products with cytotoxic activity were reported in the literature [126] with the majority of compounds only tested for cytotoxicity in cell culture assays. Although cytotoxic activity is regarded as the first indicator in identifying anticancer drugs [127], we have considered these compounds to be cytotoxic unless further experimentation indicates their potential role as an anticancer drug. Increasing evidence has shown that cell death can be induced via three different mechanisms: apoptosis, autophagy and oncosis [128]. Most of these sponge-derived novel compounds have been screened for cytotoxic activity but not for apoptosis [129,130,131], although analysis should focus on a number of cancer relevant targets associated with the cell cycle, signal transduction, angiogenesis or apoptosis [132,133,134,135]. A recent overview (2011) retrieved scientific papers identifying 39 compounds from marine sponges with apoptosis-inducing anticancer properties [136]. In another example that distinguishes cytotoxicity from antitumor activity, a recent study used a novel *in vitro* assay to screen 2036 extracts from 683 individual sponges that led to the identification of bioactive compounds (which were prepared in pure form and in sufficient quantities) that could treat solid tumors [137].

**Figure 7:**
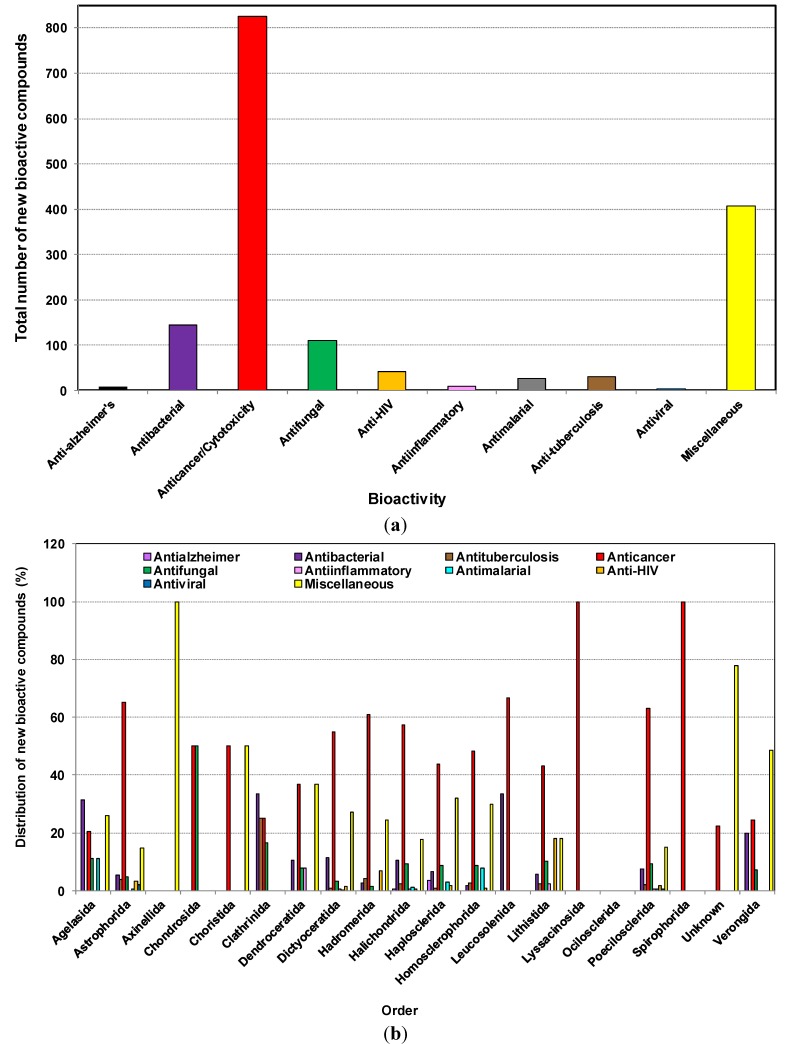
(**a**) Total number of new compounds isolated from different marine sponges with various bioactivities from 2001 to 2010; (**b**) The distribution of new compounds isolated from different orders of marine sponges with various bioactivities from 2001 to 2010.

Figure 7a,b show that the number of compounds with reported inhibition of cancer cell lines (or cytotoxicity) was highest with the number of 825, antibacterial activity at 145, and antifungal and anti-HIV activities at 111 and 41, respectively. Thus, cytotoxicity or anticancer activity contributed at least half of the reported activity. Progress towards marine anticancer drugs dominates, with the prime source phylum being sponges, followed by microorganisms, tunicates and mollusks [22]. Antiviral and anti-HIV activities have been observed from samples of Astrophorida*,* Lithistida and Poecilosclerida. The most promising antiviral substances from sponges appear to be 4-methylaaptamine, manzamines [19], besides Papuamides C and D [138], haplosamates A and B [139] and avarol [140] which are examples of HIV-inhibiting compounds from different sponges.

In a recent overview of 132 natural products from marine sources obtained during the period 2002–2011, which exhibited anti-HIV activity, it was reported that sponges contributed more than half of all anti-HIV natural products from marine organisms. These were mainly alkaloids and cyclic depsipeptides [141], such as Cortistatin A (CA), a recently discovered natural steroidal alkaloid isolated from the marine sponge *Corticium simplex* [142]. It has been reported to display anti-proliferative properties towards human umbilical vein endothelial cells (HUVECs) with an average half-maximal inhibitory concentration (IC_50_) of 0.35 μM [142,143]. A recent study showed that Cortistatin A potently suppresses Tat-dependent HIV transcription [144]. Further reading on the structural characteristics of sponge derived cyclodepsipeptides can be found in a recent review [145]. It is important to note that Halichondrida and Haplosclerida are the only orders from which anti-Alzheimer’s activity was reported during the last decade.

There have been many interesting compounds with unusual structures with potential activity which have been observed during the last decade. Some of the compounds are illustrated in Table 3.

**Table 3:** Selected compounds with unusual structure and significant activity from sponges.

Organism	Order	Compound Name	Chemical Class	Special Feature/Activity	Source, Country, Year,/Depth	Reference
*Sarcotragus* sp.	Dictyoceratida	Sarcotragin A, & B	Trisnorsesterterpenoid lactam	Showed moderate cytotoxicity (LC_50_ 207 μg/mL) toward the leukemia cell-line K562	Seoguipo, Jaeju Island, Korea, 2001	[146]
*Polymastia tenax*	Hadromerida	5α,6α-epoxy-24R*-ethylcholest-8(14)-en-3β,7α-diol and 5α,6α-epoxy-24R*-ethylcholest-8-en-3β,7α-diol	Sterol	Exhibited significant cytotoxic activity *vs.* human lung carcinoma (A-549), human colon carcinomas (HT-29 and H-116), and human prostate carcinoma (PC-3) cell lines with the LC_50_ (μg/mL) value of 5–10, 1–5, 1–5, 0.5–1 and 1–5	Punta de Betín, Bahía de Santa Marta, in the Colombian Caribbean, Colombia, 2002	[147]
*Crella spinulata*	Poecilosclerida	Benzylthiocrellidone	Bis-dimedone thioether	First report of a natural product containing a dimedone moiety. No activity reported	Davies and Bowden Reefs Australia, 2002	[148]
*Ectyoplasia ferox*	Poecilosclerida	Ectyoceramide	Galactofuranosylceramide (GSL)	The first example of a monohexofuranosylceramide and the first natural GSL with its first sugar in the furanose form. No activity reported	Island of Rum Cay, Bahamas, 2000	[149]
*Cribrochalina olemda*	Haplosclerida	Kapakahine E	Peptide (cyclic)	Kapakahine E showed moderate cytotoxicity against P388 murine leukemia cells at IC_50_ of 5.0 μg/mL	Pohnpei, Micronesia, 2003	[150]
*Haliclona Viscosa*	Haplosclerida	Viscosamine	Trimeric 3-alkyl pyridinium alkaloid	First trimeric 3-alkyl pyridinium compound from a marine environment. No activity reported	Coast of Blomstrandhalvøya, near Hansneset, Kongsfjorden, Arctic Ocean, 2003	[151]
*Phakellia fusca*	Axinellida	Compound 1, 2, 3	5-Fluorouracil alkaloid	First report of fluorine containing natural products from a marine source. No activity reported	Yongxiong Island of the Xisha Islands, South China Sea, China 2003	[152]
*Agelas clathrodes*	Agelasida	Clarhamnoside	Rhamnosylated *R*-Galactosylceramide	The first Rhamnosylated *R*-Galactosylceramide, a glycolipid containing an unusual l-rhamnose unit. No activity reported	Grand Bahamas Island (Sweetings Cay), Bahamas, 2004	[153]
*Psammocinia* sp.	Dictyoceratida	Psymberin	Cytotoxin (distantly related to the Pederin family)	Several melanoma, breast, and colon cancer cell lines demonstrated high sensitivity (LC_50_ < 2.5 × 10^−9^ M) to psymberin, and all six leukemia cell lines proved comparably insensitive	Papua New Guinea, 2004	[154]
*Callyspongia abnormis*	Haploscerida	Callynormine A	Cyclic Peptide	Represents a new class of heterodetic cyclic peptides (designated endiamino peptides). This compound possessing an α-amido-β-aminoacrylamide cyclization functionality	Shimoni reef, Kenya, 2004	[155]
*Axinella infundibula*	Halichondrida	Axinelloside A	Lipopolysaccharide (Sulfated)	Axinelloside A, a complex polysulfated glycolipid, which strongly inhibited the activity of human telomerase with an IC_50_ value of 0.4 μM	Shikine-jima Island, the Izu Islands, Japan, 2005	[156]
*Theonella swinhoei*	Lithistida	Plytheonamide A, B	Polypeptide	Showed cytotoxicity against P388 murine leukemia cells with IC_50_ values of 78 and 68 pg/mL, respectively. Linear polypeptides with unprecedented structural features	Hachijo-jima Island, Japan, 2005	[157]
*Neopetrosia* sp.	Haplosclerida	Neopetrosiamide A, B	Peptide (diastereomeric tricyclic)	Active in inhibiting the amoeboid invasion by human tumor cells	Near Milne Bay, Papua New Guinea, 2005	[158]
*Prianos osiros*	Haplosclerida	(3 *R*,3′*R*,5*S*)-3,3′,5,19′-tetrahydroxy-7′,8′-didehydro-γ,ε-carotene-8-one	Acetylenic carotenoid	Contains an unusual cytotoxic carotenoid	Pohnpei, Micronesia, 2005	[159]
*Ircinia* sp.	Dictyoceratida	Irciniasulfonic acid B	Fatty acid derivative (taurine conjugated)	Reversed the multi-drug resistance to vincristine in KB/VJ300 cells at the concentration of 100 μM	Tsuzumi Island, Fukuoka Prefecture, Japan, 2006	[160]
*Suberites japonicus*	Hadromerida	Seragamide A–F	Depsipeptide (actin targeting)	Caused multinuclei formation in cells at 0.01–0.02 μg/mL	Seragaki, Okinawa, Japan, 2006	[161]
*Theonella swinhoei*	Lithistida	Hurghadolide A	Macrolide	Caused disruption of the actin cytoskeleton at concentrations of 7.3 nM. Active against *Candida albicans* (MIC 31.3 μg/mL)	Red Sea, Egypt, 2006	[89]
*Theonella swinhoei*	Lithistida	Swinholide I	Macrolide	as above	Red Sea, Egypt, 2006	[89]
*Coelocarteria* cfr.* singaporensis*	Poecilosclerida	Coelodiol and Coelic acid	Diterpene (ent-isocopalane)	Inhibit the *in vitro* growth of MKN-45 cell line (human gastric adenocarcinoma) at 20 and 40 μg/mL respectively	Bunaken, Marine Park (North Sulawesi), Indonesia, 2006	[162]
*Lendenfeldia* sp.	Dictyoceratida	( *S*)-2,2′-Dimethoxy-1,1′-binaphthyl-5,5′,6,6′-tetraol	Naphthalene dimer	Significantly inhibited both hypoxia-induced (IC_50_ values 4.3 µM) and iron chelator (1, 10-phenanthroline)-induced HIF-1 activation in T47D breast tumor cells. This compound inhibited HIF-1 activation at concentrations that were significantly lower than those that suppressed tumor cell viability	Collected at 2 m depth on May 22, 1993 (sample C011337), from a sea grass bed, Indonesia, 2007	[163]
*Erylus formosus*	Astrophorida	Eryloside F1–F4	Triterpene glycoside	At a concentration of 100 μg/mL were found to activate Ca2 influx into mouse spleenocytes. biosides having aglycons related to penasterol with additional oxidation patterns in their side chains	Puerto Morelos (the Caribbean Sea), Mexico 2007	[164]
*Erylus formosus*	Astrophorida	Eryloside M–Q	Triterpene glycoside	As above, contain new variants of carbohydrate chains with three, four and six sugar units. Contain 14-carboxy-24-methylenelanost-8(9)-en-3β-ol	Puerto Morelos (the Caribbean Sea), Mexico, 2007	[164]
*Cacospongia mycofijiensis*	Dictyoceratida	CTP-431	Thiopyrone	Showed only mild cytotoxicity (IC_50_: 18 μM) against human colon carcinoma HCT-116. This compound has no previous precedent in natural products chemistry. Its structure including absolute configuration as 8*R*,9*R*,10*S*,13*S*	Beqa Lagoon, Fiji, 2008	[165]
*Homophymia* sp.	Lithistida	Homophymine A	Cyclodepsipeptide	Exhibited cytoprotective activity against HIV-1 infection with a IC_50_ of 75 nM	Coast of New Caledonia, 2008	[166]
*Ianthella* sp.	Verongida	Petrosterol-3,6-dione and 5α,6α-epoxy-petrosterol	C29 sterol	Showed growth-inhibitory effects with IC_50_ values of 8.4, 19.9, 17.8, 16.2 and 22.1 μM against lung (A549), colon (HT-29), breast (MCF-7), ovary (SK-OV-3), and two types of leukemia (HL-60 and U937) human cancer cell lines	Namyet Island, Khanh Hoa province, Vietnam, 2009	[167]
*Topsentia* sp.	Halichondrida	Geodisterol-3- *O*-sulfite and 29-demethylgeodisterol-3-*O*-sulfite	Sterol (sulphated)	Reverses efflux pump mediated fluconazole resistance. Also enhances fluconazole activity in a *Saccharomyces cerevisiae* strain overexpressing the *Candida albicans* efflux pump MDR1, as well as in a fluconazole-resistant *Candida albicans* clinical isolate known to overexpress MDR1	Chuuk, Micronesia, 2009	[168]
*Spongia* *(Heterofibria)* sp.	Dictyoceratida	Heterofibrin A1–A3 and B1–B3	Fatty acid	Possess a diyne-ene moiety, while the monolactyl and dilactyl moiety featured in selected heterofibrins is unprecedented in the natural products literature. Inhibited lipid droplet formation in A431 fibroblast cells (up to 60% at 10 μM)	Great Australian Bight, Australia, 2010	[169]
*Xestospongia* sp.	Haplosclerida	Xestosaprol F–M	Xestosaprol (pentacyclic compound)	Showed moderate inhibition of the aspartic protease BACE1 (memapsin-2), which has a central role in the etiology of Alzheimer’s disease with the IC_50_ value of 135 ± 11 μM. First examples of a monooxygenated A-ring	Coral reef at Sangalaki, Indonesia, 2010	[170]
*Theonella swinhoei*	Lithistida	Paltolides A–C	Peptides (Anabaenopeptin like)	Closely related to a group of anabaenopeptins that are submicromolar inhibitors of carboxypeptidase U with greater than 50 fold selectivity over other carboxypeptidases	Uchelbeluu Reef, Palau, 2010	[171]
*Neopetrosia proxima*	Haplosclerida	Neopetrosiamine A	Alkaloid (tetracyclic bis-piperidine)	Exhibited strong inhibitory activity against MALME-3M melanoma cancer, CCRF-CEM leukemia, and MCF7 breast cancer with IC_50_ values of 1.5, 2.0, and 3.5 μM, respectively. *In vitro* activity *vs.* pathogenic strain of *Mycobacterium tuberculosis* (H37Rv) and *Plasmodium falciparum*	Mona Island, Puerto Rico, 2010	[172]
*Iotrochota baculifera*	Poecilosclerida	Baculiferins A–O	*O*-sulfated pyrrole alkaloids	Baculiferins C, E–H, and K–N (4, 6–9, 12–15) are potent inhibitors of HIV-1 IIIB virus in both MT4 and MAGI cells. Additionally could bind to the HIV-1 target proteins Vif, APOBEC3G, and gp41	Inner coral reef, Hainan Island, China, 2010	[173]

### 2.8. Distribution of New Compounds Based on Country/Geographical Area

One important aspect of sponges is their geographical location. A high percentage of bioactive sponge species were reported from different geographical regions [174,175,176].

**Figure 8:**
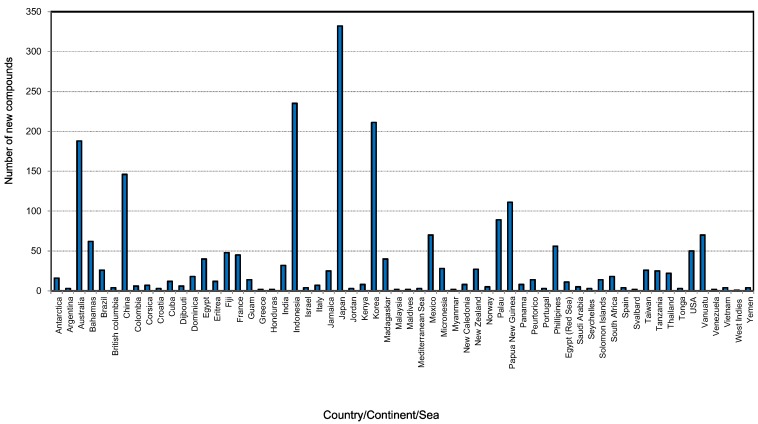
Total number of new compounds isolated from different marine sponges and their source locations from 2001 to 2010.

Figure 8 shows that the new compounds were mainly isolated from sponges collected from 61 countries. Of these, sponges from Japan had the highest number of compounds (332) followed by Indonesia (235), Korea (211). Sponge samples from Australia and China contributed 187 and 146 compounds, respectively, during the last decade. The Bahamas, Mexico, Palau, Papua New Guinea, Philippines and Vanuatu are the other countries which were the source of more than 50 new compounds found in the last ten years. The obvious question is why do some regions show a high diversity of compounds as well as an abundance of sponges? Studies showed that the highest concentration of toxic or antioxidant sponge metabolites are found in habitats such as coral reefs that are characterized by intense competition and feeding pressure from carnivorous fish [177]. The adaptive significance of the chemical defenses of sponges are that they are highly effective against most species of fish and a group of shell-less gastropods, the nudibranchs that feed on sponges and sequester their chemical armory [177]. An excellent paper on the global diversity of sponges includes global sponge diversity information which was collected from different regional projects and resources and also reviewed was information on invasive sponges that might well have some influence on distribution patterns in the future [83].

**Figure 9:**
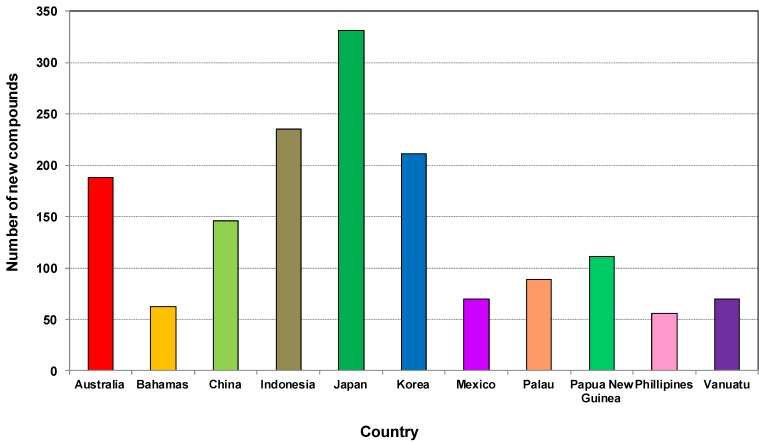
Total number of new compounds isolated from marine sponges from the top 10 source countries from 2001 to 2010.

Figure 9 shows the total number of new compounds isolated from the top ten countries. It has been found that each year from 2001 to 2010 at least 20 new compounds were isolated from Japanese sponge samples, with the highest output occurring in 2004 and 2005 with 46 new compounds (Figure S2, Supplementary Information). From 2006 to 2009 at least 20 compounds were found each year in Indonesia. Marine invertebrates, which are plentiful in the Indo-Pacific regions including Indonesia, are rich in secondary metabolites and are becoming targets for the continuing search for bioactive compounds [178].

Although in 2001 the highest contributors of new compounds derived from sponges were from Japan, Korea and Australia at 22.9%, 18.9% and 14.3%, respectively, the scenario changed during 2010 where the highest contributions of new compounds came from China, Australia and Indonesia at 23.9%, 21.3%, and 21%, respectively [179]. China started its marine high-tech program since 1996, and after 15 years it emerged as one of the highest contributors to marine sponge natural products discovery in 2010. This achievement demonstrates how significant the government science and technology funding policy can impact the development of marine biotechnology. Australia is one of only 17 recognised megabiodiverse countries primarily based on its highly biodiverse and endemic terrestrial flora and fauna [180,181]. While the full extent of Australian marine biodiversity remains relatively unexplored [182], several marine biodiversity hotspots including centers of endemicity have been recognized, especially in coral reefs [183,184], the temperate coastline [185] and the Great Australian Bight off the coast of South Australia [186]. There have been reports on the high species diversity of sponges in the north west [187,188,189,190], in the deep sea off the south west [191,192] of the Bight, and the Great Barrier Reef [189]. Therefore, there is urgent need to explore those potential locations in order to obtain new compounds and drugs for the future.

The Australian Institute of Marine Science (AIMS) conducted a study based on the Australian marine habitat and identified biogeographic bioactivity hotspots that correlated with biodiversity hotspots. AIMS found that high-level phylogeny, and therefore the metabolic machinery available to an organism, is a major basis of bioactivity, while habitat diversity and ecological circumstance are possible drivers in the stimulation of this machinery and bioactive secondary metabolism [193]. Therefore, in near future, knowledge of metabolomics coupled with genomics tool and bioinformatics could be a high level device for the exploration of target specific bioactive compounds from sponges.

In addition, microbes associated with marine sponges could vary with the geographical area, so, if these associated bacteria are responsible for producing compounds, then it is possible that the same sponge species in different geographical locations could produce different secondary metabolites [194,195]. However, a recent study showed the stability of bacterial communities in two temperate sponges exposed to environmental variation, which is consistent with previous research on other temperate sponges. This study used next generation sequencing and revealed how different components of bacterial communities associated with *Ecionemia alata* and *Tethya* *bergquistae* responded to environmental variation *in situ* [196]. The similarity observed in bacterial communities among specimens occupying different habitats suggests that environmental variation occurring in those habitats does not affect the stability of the community, and hence, most likely does not radically alter the metabolism of these sponges. The study recommends further study to improve the understanding of the role of microbial symbiont communities which may affect the physiology and ecology of sponges on temperate rocky reefs [196].

A recent pyrosequencing analysis of 32 sponge species from eight locations around the world identified few bacterial species that are common to more than a handful of sponge species [177]. They stated that different sponges were found to contain different bacterial species (species-specific community) but share only very few bacterial species (core community) [197]. However, the bacterial species in different sponges are still more closely related to each other than, for example, to seawater bacteria (indicated by Plus-OTUs and sponge-specific clusters), consistent with previous studies suggesting at least partially overlapping communities among different sponges. Sponges therefore contain a uniform, sponge-specific bacterial community although each sponge species contains different bacterial species [197]. Perhaps, this is one of the main reasons for getting new compounds in species level regardless of the genera and order. Although we commented on this, based on our study of sponges of the last decade, further study to prove this hypothesis would be worthwhile.

## 3. Conclusions

In the decade 2001–2010 marine sponges continued to be the most promising source of marine natural products. Marine microorganisms and phytoplankton grew rapidly to become the third largest source by increasing their contribution from 9% to 39% during the decade. Sponges are a reservoir of marine microorganisms with up to 40%–60% as microbial biomass. In the coming decade sponge associated microorganisms promise to be an outstanding source of the NMNP.

From the current trend of discovery it could be predicted that China, Australia and Indonesia will be the source of more new compounds in the future competing with Japan and Korea. However, although Indonesia is an excellent source of sponges, most of their studies were conducted by scientists in other countries. Certainly, exploration of new compounds from marine sponges is dependent on government funding and policy, industrial interest and investment, research facilities, the expertise of scientists, infrastructure and laboratory facilities, equipment, machinery and institutional support.

Astrophorida, Dictyoceratida, Halichondrida, Haplosclerida and Poecilosclerida were the key orders of sponges studied during the first decade of the 21st century. The examination of the contribution from an individual species revealed that regardless of the order each species contributed on average 3–5 compounds. The high number of new compounds was the result of the high diversity of species from these particular orders. Alkaloids (20%), terpenes (14.7%) or terpenoids (8.3%) and peptides (6.8%) represented the three main chemical classes of compounds discovered from sponges in this period, and together with the other chemical classes showed a range of biological activities. Of all the biological activities investigated cytotoxicity or anticancer activity against different cancer cell lines was most frequently reported at 53.6%. Antibacterial and antifungal activity were two other areas where new compounds showed potential activity at 9.4% and 7.2%, respectively. Anti-Alzheimer’s, antibacterial, antituberculosis, anticancer, antifungal, anti-inflammatory, antimalarial, antiviral, anti-HIV were the other activities exhibited by the new compounds from sponges. Because sponge extracts showed potent cytotoxic activity, which is often reported as anticancer activity, it is very important to study the mode of action of these extracts by isolating pure compounds rather than only testing cytotoxicity.

The order Dictyoceratida was found to be the most prolific producer of new compounds among all the sponge orders studied. *Dysidea* sp. and *Ircinia* sp. were found to be the most promising genera because of their capacity for producing new bioactive compounds.

In any event, the discovery of marine natural products from sponges relies particularly on finding new genera and species from the most prolific to the least abundant orders, which is still achievable. In order to overcome the problem of production of sponge-derived compounds, synthesis of bioactive, low molecular weight compounds by cloning biosynthetic gene clusters using recombinant techniques could be applied. Most importantly, sponge derived compounds should be utilized with a combination of innovative technologies which could develop new fields of application that will impact significantly on biotechnology.

## Supplementary Files

Supplementary File 1
