# Supplementary material for: Marine Sponge Derived Natural Products between 2001 and 2010: Trends and Opportunities for Discovery of Bioactives
Source: Mar Drugs. 2014 Aug 19;12(8):4539–77. doi: 10.3390/md12084539 (PMC4145330; doi:10.3390/md12084539)

Supplementary Information

Figure S1. Average number of new compounds per species isolated from different orders of marine sponges from 2001 to 2010.

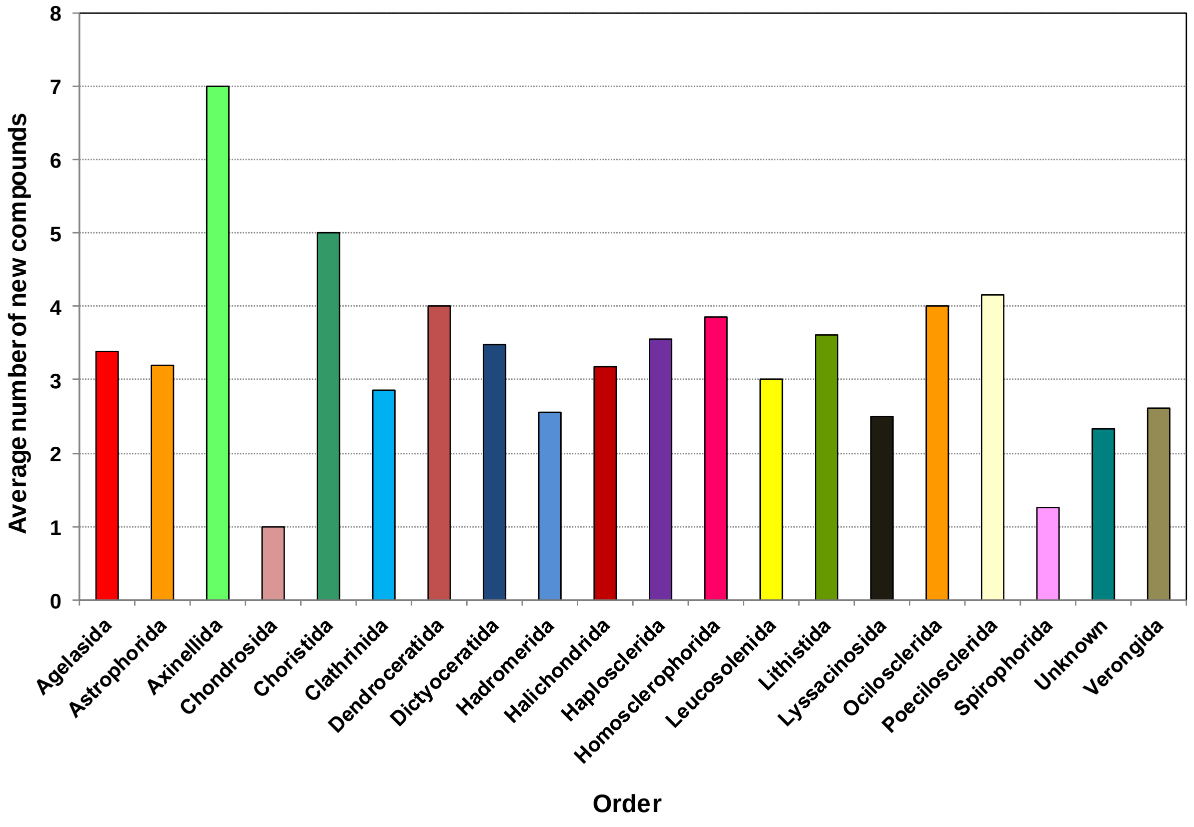

Figure S2. The distribution of new compounds isolated from different marine sponges from the top 10 countries in the world from 2001 to 2010.

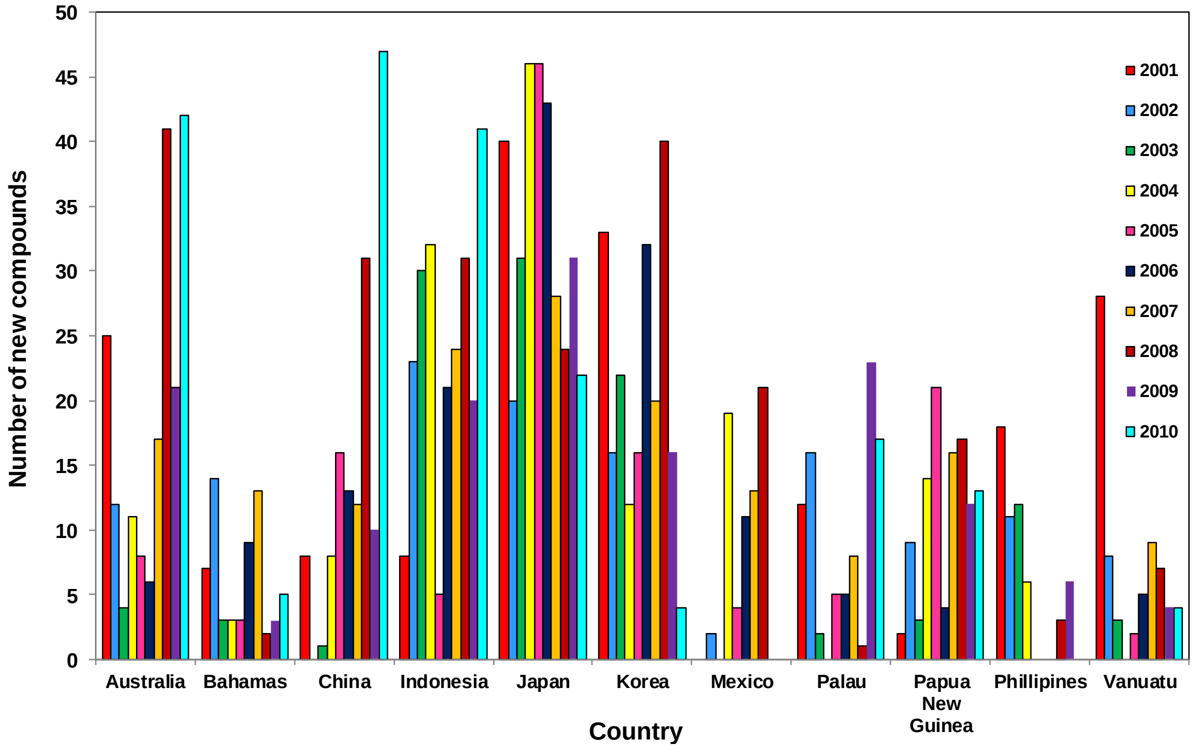

Supplement: Supplementary File 1 [file marinedrugs-12-04539-s001.pdf]
